# Epidemiology of Vascular Access-Associated Infections in Hemodialysis: A Single-Center Retrospective Study in Tbilisi, Georgia (January 2022–January 2025)

**DOI:** 10.7759/cureus.94959

**Published:** 2025-10-20

**Authors:** Tamar Kasradze, Tamar Didbaridze, Irma Tchokhonelidze

**Affiliations:** 1 Nephrology, Tbilisi State Medical University and Ingorokva High Medical Technology University Clinic, Tbilisi, GEO; 2 Internal Medicine, Tbilisi State Medical University, Tbilisi, GEO; 3 Microbiology, The First University Clinic of Tbilisi State Medical University, Tbilisi, GEO; 4 Microbiology, Tbilisi State Medical University, Tbilisi, GEO

**Keywords:** arteriovenous fistula, catheter-related bloodstream infection, central venous catheter, hemodialysis, incidence rate ratio, kaplan–meier, logistic regression

## Abstract

Background

Infections related to vascular access (VA) remain one of the most serious complications in hemodialysis (HD), contributing substantially to patient morbidity and mortality. Central venous catheters (CVCs) confer a substantially higher infection risk compared to arteriovenous fistulas (AVFs), but local epidemiological data are limited.

Objective

We aimed to investigate the incidence of VA-related infections by access type (CVC vs. AVF, using access-days) and to identify risk factors, with secondary outcomes including infection-related hospitalization and mortality, in HD patients at the largest single center in Tbilisi, Georgia (2022-2025).

Methods

A retrospective cohort study was conducted on 386 adult HD patients from January 2022 to January 2025. Demographic, clinical, and VA data were analyzed. Cox proportional hazards and logistic regression models assessed associations between VA type, diabetes, and infection or hospitalization, accounting for person-time at risk. Kaplan-Meier curves compared time-to-infection, and incidence rates were calculated per 1,000 access-days with corresponding incidence rate ratios (IRRs) and attributable fractions.

Results

Out of 386 patients, 208 (53.9%) used CVCs and 178 (46.1%) used AVFs. A total of 116 (30.1%) patients developed bacterial infections, of which 69 (59.5%) were access-related. The infection rate was markedly higher among CVC users compared with AVF users (0.731 vs. 0.042 infections per 1,000 access-days; incidence rate ratio (IRR) = 17.25; 95% confidence interval (CI): 8.21-41.79; p < 0.001). The incidence rate difference was 0.689 infections per 1,000 access-days (95% CI: 0.50-0.88; p < 0.001), with an attributable fraction among the exposed of 94.2% (95% CI: 87.8-97.6).

In the univariate Cox regression analysis, CVC use was strongly associated with an increased hazard of bloodstream infections (BSIs) (hazard ratio (HR) = 19.18; 95% CI: 8.72-42.23; p < 0.001) and infection-related hospitalization (HR = 4.10; 95% CI: 2.50-6.71; p < 0.001). Diabetes mellitus was not significantly associated with BSIs (HR = 0.91; 95% CI: 0.55-1.51; p = 0.715) but showed a weak association with hospitalization (HR = 1.04; 95% CI: 1.00-1.07; p = 0.041). Age was not significantly related to either outcome. Infection-related mortality occurred in 23 (19.8%) patients.

Conclusions

Using CVCs is a significant and modifiable risk factor for VA-related infections in HD patients. Strategies to reduce CVC use focus on timely AVF placement, and strengthening infection control protocols is essential to decrease infection-related morbidity and mortality.

## Introduction

Infections remain the second leading cause of hospitalization and death among hemodialysis (HD) patients, surpassed only by cardiovascular diseases [1,2]. Vascular access (VA)-related infections, defined as infections occurring at the site of a central venous catheter (CVC), arteriovenous graft (AVG), or arteriovenous fistula (AVF) and often leading to bacteremia, account for the majority of these cases. VA-related infections, including catheter-related bloodstream infections (CRBSIs) and infections of AVGs, account for the majority of these cases. By contrast, the use of AVFs is associated with a substantially lower risk of infection. VA-related infections are a leading cause of sepsis [3] and endocarditis [4] in HD patients. Notably, the use of AVFs is associated with a significantly lower risk of infection compared to patients initiating HD with CVCs or AVGs [3].

While CRBSIs are widely recognized for their high incidence and severe outcomes, infections of AVFs, although less frequent, still contribute significantly to morbidity, hospitalization, and high healthcare expenditure [5,6]. Published data suggest that the incidence of CRBSI ranges from 1.1 to 5.5 episodes per 1,000 catheter-days, whereas AVF-related infections occur at far lower rates, often <0.1 episodes per 1,000 fistula-days [7,8,9,10]. This disparity underscores the importance of prioritizing AVF creation whenever feasible, consistent with the Kidney Disease Outcomes Quality Initiative (KDOQI) guidelines, which recommend establishing AV access - either AVF or AVG - for patients requiring HD, when aligned with their kidney failure (KF) life plan and overall goals of care [11]. Nevertheless, CVCs remain widely used, particularly in patients referred late for dialysis initiation, despite their substantially increased risk of infection.

Patient-specific factors also influence infection risk. Diabetes mellitus is a common comorbidity among HD patients and is well recognized as being associated with increased susceptibility to infection [12]. However, evidence on its role in VA infections remains controversial. Some studies have reported higher rates of BSIs and hospitalizations among diabetic HD patients [13], while others found no significant association after adjusting for VA type and other confounders [14]. These findings suggest that the kind of VA may exert a more substantial effect on infection risk than diabetes itself, although further investigation is warranted.

This retrospective epidemiological study aims to raise clinical awareness about infections among HD patients in Georgia. The primary objective is to determine the rate of VA-associated infections and identify potential risk factors that lead to hospitalization and mortality. Furthermore, this research builds upon previous studies on VA by incorporating information on infection rates.

The findings of this study have important epidemiological and clinical implications, particularly for identifying common risk factors of VA infections and developing strategies to reduce infection-related hospitalizations and mortality. These results may ultimately contribute to the development of improved infection control protocols and better patient outcomes in HD centers.

## Materials and methods

This study is a retrospective observational analysis of a cohort of 386 patients undergoing maintenance HD. Data were collected and analyzed over a three-year period, from January 1, 2022, to January 1, 2025, in a hospital-based dialysis center, Tbilisi, Georgia. The study was conducted in collaboration with Tbilisi State Medical University and Ingorokva High Medical Technology University clinic, Tbilisi, Georgia.

Study population

This study involved 386 adult patients with KF who underwent chronic HD through permanent CVCs and AVFs. Data from the patients were collected using a standard form that included demographic information, diagnoses, infection records, hospitalization rates, causes of KF, and comorbidities.

The study cohort was incident and included adult patients who were already receiving maintenance HD with a functioning VA at the beginning of the study period. The time zero for follow-up was defined as the date of each patient’s first dialysis session within the study window (January 1, 2022-January 1, 2025). Patients were followed until the occurrence of a VA-related infection, death, transfer to another dialysis center, or the end of the observation period, whichever came first.

CRBSI was defined according to the Centers for Disease Control and Prevention (CDC)/National Healthcare Safety Network (NHSN) and Infectious Diseases Society of America (IDSA) criteria, requiring both clinical signs of infection (such as fever, chills, or local catheter site inflammation) and microbiological confirmation with positive blood cultures and/or catheter tip culture. AVF infection was defined as the presence of localized inflammatory signs (erythema, warmth, tenderness, or purulent drainage) with or without bacteremia. Multiple infections per patient were included if they fulfilled diagnostic criteria and represented distinct clinical episodes. Case adjudication was performed through detailed review of patient charts by the study investigators; however, blinding of adjudicators was not feasible due to the retrospective design.

Participants were selected based on specific inclusion and exclusion criteria. They were grouped according to demographic characteristics and health conditions, including age (adults aged 18 years and older) and comorbidities. In addition, we considered treatment-related factors that could affect infection risk and long-term health outcomes, such as the duration of HD- and KF-related medical complications.

Patients with CVCs were divided into three groups and received one of three catheter-locking solutions: sodium bicarbonate (8.4%), citrate (2.2%) combined with gentamicin (0.5 mg/mL), or unfractionated heparin. The type of locking solution was assigned according to the dialysis shift. All measures were implemented alongside strict surveillance protocols to ensure early detection and management of catheter-related infections.

All patient charts were carefully reviewed and verified prior to inclusion, and no missing data were present for key outcome variables such as infection and hospitalization; therefore, no data imputation was required.

The study received approval from the Institutional Review Board (IRB) of Ingorokva High Medical Technology University Clinic (approval no. 3/2025/02, dated February 2, 2025), and the requirement for written informed consent was waived. Eligibility criteria included being 18 years or older, undergoing chronic HD treatment, and having a minimum of 75 days of follow-up. Patients aged 17 years or younger were excluded from the study.

Data collection

The database included the following variables: patient ID, date of birth, cause of KF, date of VA creation or CVC placement, and date of VA-related infection. In addition, it included outcomes of antibiotic therapy among those who developed infections and those admitted to the hospital.

Statistical analysis

Descriptive Analysis

Numerical variables, such as age and duration on dialysis, were summarized using the mean with standard deviation (SD) and the median with interquartile range (IQR). Categorical variables, such as catheter access, development of infection, hospitalization, and recovery from infection, were described using percentages.

Association Analysis

Age, diabetes, and VA were treated as exposure variables, while infection development and hospitalization due to infection were considered outcome variables.

Age was analyzed as a continuous variable. Diabetes (both type I and type II) was grouped and compared with all other underlying conditions, forming a binary variable. Similarly, fistula access was compared to CVC access as another binary exposure variable. Both outcome variables - development of bacterial blood infection and hospitalization due to infection - were binary

The association between the exposure variables and bacterial infection was analyzed with Cox regression.

Person-time at risk was calculated for four categories: 1) patients who developed catheter-related blood infection (person-time at risk was calculated from the implementation of the catheter till the development of infection or till the observation time/ removal of the catheter/death); 2) patients who did not develop catheter-related blood infection (person-time was calculated from implementation of the catheter till the end of observation time/removal of the catheter/death); 3) patients with fistula and no bacterial blood infection (person-time at risk was calculated from the date of inclusion in the study till the end of observation period or death); and 4) patients with fistula and bacterial blood infection (person-time at risk was calculated from the date of inclusion in the study till the development of blood infection).

The association between hospitalization due to infection and exposure variables was analyzed with univariate logistic regression.

In addition, Kaplan-Meier survival curves were generated separately for diabetic versus non-diabetic patients and for AV fistula versus CVC access to compare the time to infection occurrence over the entire follow-up period. Incidence rate ratios (IRRs) and attributable fractions were also calculated to assess the impact of removing the exposure. To account for variations in exposure time, infection incidence was calculated separately for catheters and AVFs and expressed as events per 1,000 access days. CRBSI rates were defined as the number of CRBSI episodes per 1,000 catheter-days. By contrast, AV fistula infection rates were defined as the number of AV fistula-related infections per 1,000 fistula-days. This approach enabled a standardized comparison of infection risk between the different types of VA.

Variables showing a statistically significant association in univariate analysis (logistic or Cox) (p < 0.05) were subsequently included in a multivariable logistic regression model.

Exposure transitions between VA types (e.g., from CVC to AVF) were not modeled as time-varying exposures. Multiple infections per patient were included, provided they fulfilled diagnostic criteria and were considered distinct clinical episodes.

## Results

The dataset consisted of 386 HD patients. The minimum age was 18, and the maximum age was 93. The mean age was 56 years (SD 14.8), and the median age was 58 years (IQR 47-66). The mean duration of follow-up was 929 days (SD 262); the median was 1,096 days (IQR 761-1096), with a minimum of 75 days and a maximum of 1,096 days. Of the 386 patients, 208 (53.9%) had CVC access, and 178 (46.1%) had AVFs. The most common underlying cause of kidney failure among the 386 patients was hypertension in 151 (39.1%), followed by type II diabetes in 146 (37.8%), polycystic kidney disease (PKD) in 22 (5.7%), glomerulonephritis in eight (2.1%), and other causes in 59 (15.3%).

In total, 116 (30.1%) patients developed bacterial infections, of whom 107 (92.2%) were hospitalized. Among the 116 infection cases, 69 (59.5%) were VA-related bloodstream infections (CRBSIs, 61/69 (88.4%) and AV fistula infections, 8/69 [11.6%]), and 47 (40.5%) were non-VA-related infections. The most prevalent cause among non-VA-related infections was bacterial pneumonia in 25 (53.2%), followed by urinary tract infection in eight (17.0%). Out of those with VA-related infection (n = 69), 10 (14.5%) died, 45 (65.2%) responded favorably to treatment, and 14 (20.3%) failed to respond to antibiotic therapy and subsequently required CVC removal (Table 1).

**Table 1:** Outcomes of antibiotic therapy in patients with vascular access–related infections (n = 69) Data are presented as n (%).

Outcome	N	%
Resistant to antibiotic therapy	14	20.3
Good response to antibiotic therapy	45	65.2
Fatal outcome	10	14.5

The type of VA demonstrated the strongest association with both outcomes. Patients with a CVC had a markedly higher risk of developing bloodstream infections compared to those with an AV fistula (HR = 19.18; 95% CI: 8.72-42.23; p < 0.001) and were also more likely to be hospitalized due to infection (HR = 4.10; 95% CI: 2.50-6.71; p < 0.001). Age was not significantly associated with either bloodstream infection (HR = 1.00; 95% CI: 0.99-1.02; p = 0.264) or hospitalization (HR = 1.01; 95% CI: 0.99-1.03; p = 0.109). Diabetes mellitus was not a significant predictor of bloodstream infection (HR = 0.91; 95% CI: 0.55-1.51; p = 0.715), but it showed a weak yet statistically significant association with hospitalization due to infection (HR = 1.04; 95% CI: 1.00-1.07; p = 0.041) (Table 2).

**Table 2:** Univariate association between possible risk factors and outcomes Data are expressed as hazard ratios (HR) with 95% confidence intervals (CI); HR > 1 indicates a higher risk compared with the reference group. CVC: central venous catheter; AV: arteriovenous fistula

Outcome	Risk factor	Hazard ratio	95% CI	p-value
Bloodstream infection	CVC vs. AV fistula	19.18	8.72-42.23	<0.001
	Diabetes vs. others	0.91	0.55–1.51	0.715
	Age (per year)	1.00	0.99–1.02	0.264
Hospitalization due to infection	CVC vs. AV fistula	4.10	2.50–6.71	<0.001
	Diabetes vs. others	1.04	1.00–1.07	0.041
	Age (per year)	1.01	0.99–1.03	0.109

In the multivariable logistic regression analysis assessing predictors of infection-related hospitalization, the type of VA remained a significant determinant of risk. Patients with a CVC had nearly three times higher odds of hospitalization compared with those dialyzing via an AV fistula (OR = 3.99; 95% CI: 2.43-6.56; p < 0.001), after adjusting for diabetes status. Diabetes was not significantly associated with hospitalization risk (OR = 1.03; 95% CI: 0.99-1.07; p = 0.113).

The incidence rate of bloodstream infections among patients with CVCs was 0.731 per 1,000 catheter-days, markedly higher than the 0.042 per 1,000 fistula-days observed in the AVF group (Table 3). The incidence rate ratio (IRR) was 17.25 (95% CI: 8.21-41.79), indicating a more than seventeenfold higher infection frequency in CVC users. The absolute rate difference was 0.689 infections per 1,000 VA-days (95% CI: 0.50-0.88; p < 0.001), corresponding to an attributable fraction among the exposed of 0.94 (95% CI: 0.88-0.98), confirming that the vast majority of infections were attributable to catheter use (Table 4).

**Table 3:** Incidence rates of infection by the type of vascular access Data are presented as number of events, time at risk, and incidence rates.

Measure	CVC (exposed)	AV fistula (unexposed)	Total
Failures (n)	61	8	69
Time at risk (vascular access–days)	80,715	188,760	269,475
Incidence rate (per 1,000 access-days)	0.731	0.0424	0.2486

**Table 4:** Effect estimates for incidence rate comparison Data are presented as point estimates with corresponding 95% confidence intervals. IRR = incidence rate ratio

Measure	Point estimate	95% confidence interval	p-value
Incidence rate difference (per 1,000 access-days)	0.6886	0.4998–0.8774	<0.001
Incidence rate ratio	17.25	8.21–41.79	—
Attributable fraction (exposed)	0.942	0.878–0.976	—

The infection rate among patients with diabetes was 0.1955 per 1,000 VA-days, slightly lower than the rate of 0.2038 per 1,000 VA-days among those without diabetes. The infection rate ratio (IRR) was 0.96 (95% CI: 0.56-1.60), indicating no statistically significant difference in infection risk between diabetic and non-diabetic patients. The absolute rate difference was -0.0084 per 1,000 VA-days (95% CI: -0.11 to 0.09), with a two-sided p-value of 0.8758, confirming the lack of significance. The prevalence fraction among the exposed was 4.1%, but the confidence interval included both risk reduction and increased risk, reflecting uncertainty.

**Figure 1:**
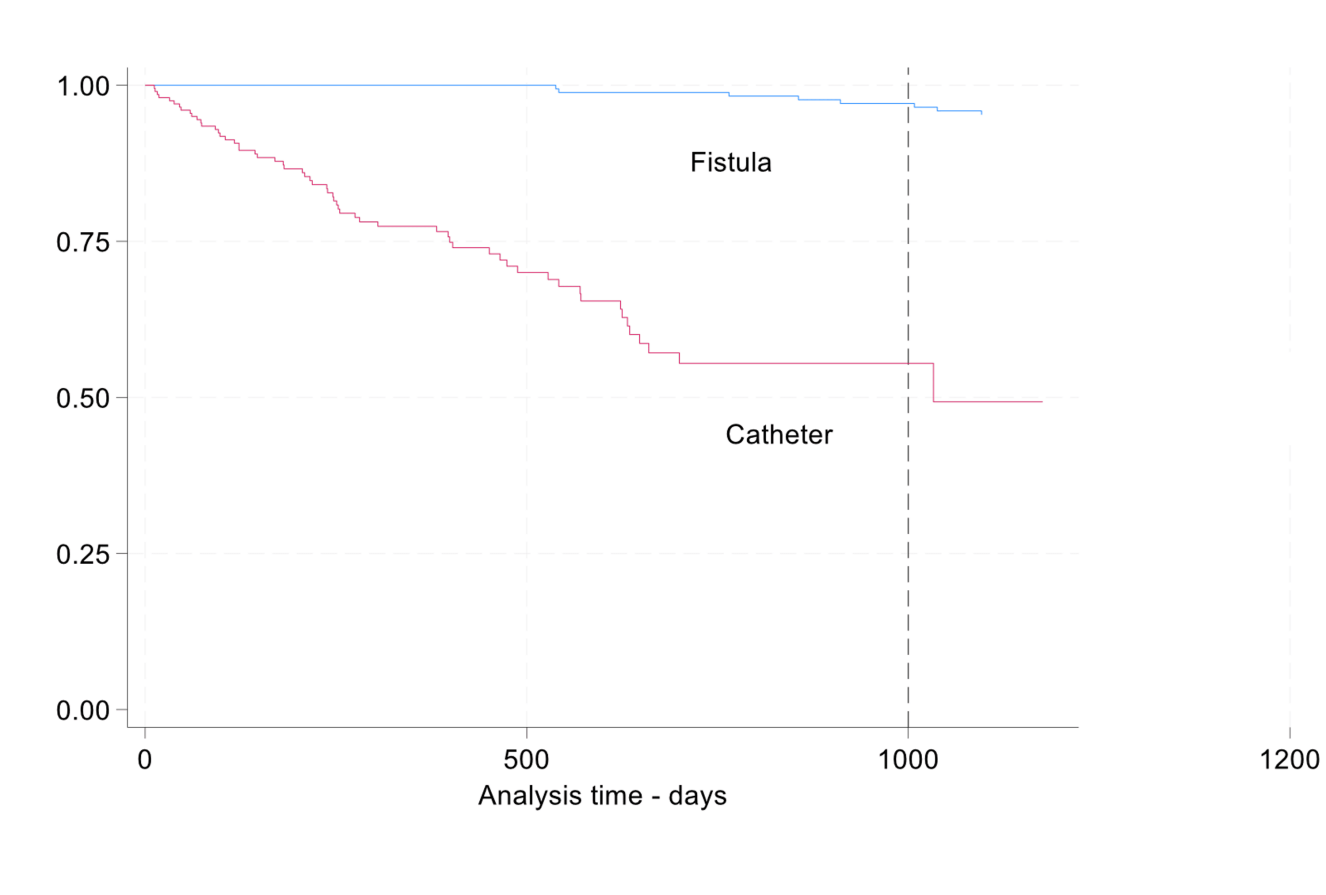
Kaplan–Meier survival estimates for the development of vascular access–related infection among patients with central venous catheters (CVC) and arteriovenous fistulas (AVF)

**Figure 2:**
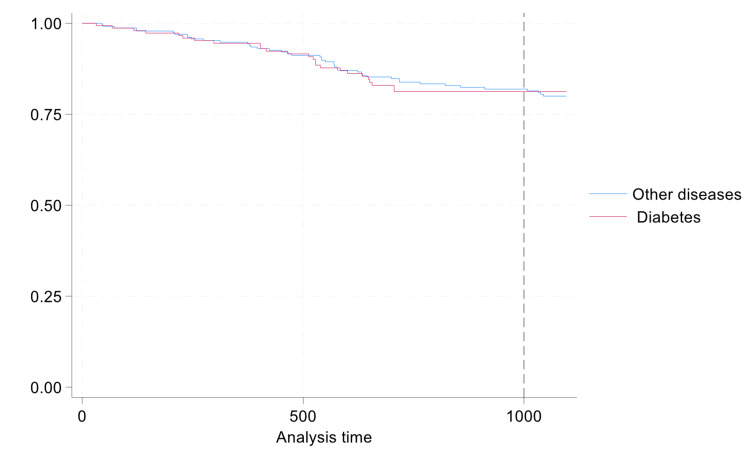
Kaplan-Meier survival estimates for developing bacterial infections among patients with diabetes and without diabetes

Figure 1 shows the Kaplan-Meier survival estimates for bacterial infection development in patients with CVCs and AVFs, highlighting the high risk in those with CVCs. Figure 2 shows Kaplan-Meier survival estimates for the development of bacterial infection among patients with and without diabetes.

## Discussion

Our single-center, retrospective observational study provides valuable epidemiological data on VA-related infections in patients undergoing chronic HD. It emphasizes the substantial burden of BSIs and related hospitalizations, particularly among patients using CVCs. Beyond confirming established evidence, our findings engage with ongoing controversies in VA practice, infection-prevention strategies, and the role of comorbidities, while also underscoring recent advances in the field.

We evaluated infection outcomes in a cohort of 386 patients undergoing HD over a mean follow-up period of over three years. The results underscore the significant impact of VA type on infection rates and clinical outcomes. Notably, 208 (53.9%) patients were dialyzed via a CVC, while 178 (46.1%) had an AVF. The findings support well-established evidence that infections remain a significant and frequent complication in the HD population, especially among patients with CVCs [15,16]. These findings are consistent with large-scale observational studies, which consistently identify catheter use as the most modifiable risk factor for infection and mortality in HD populations [17,18,19]. In our cohort, 116 (30.1%) patients developed infections; among these, 69 (59.5%) were VA-related infections (CRBSIs and AVF infections) and 47 (40.5%) were non-VA infections. The vast majority, 107 (92.2%), required hospitalization, and infection-related mortality occurred in 23 (19.8%). These observations are consistent with those of Nguyen et al. (2017) [20], who analyzed national NHSN surveillance data showing high infection-related morbidity among U.S. dialysis patients, and with Dalrymple and Go (2008) [21], who reported that infections represent a major cause of hospitalization and death in chronic kidney disease populations.

The infection rate among patients with CVCs was 0.731 per 1,000 catheter-days, which was more than 17 times higher than the rate observed among AVF users (0.042 per 1,000 fistula-days). Our comparatively lower CRBSI rate likely reflects the routine use of catheter-locking solutions (sodium bicarbonate 8.4%, citrate 2.2% with gentamicin 0.5 mg/mL, and unfractionated heparin distributed by dialysis shift) together with strict surveillance protocols. We attribute this to routine use of catheter-locking solutions (sodium bicarbonate 8.4%, citrate 2.2% with gentamicin 0.5 mg/mL, and unfractionated heparin according to dialysis shift) combined with rigorous surveillance. These findings align with the work of Fisher et al. (2020) [1], who demonstrated that antimicrobial lock solutions can significantly reduce CRBSI incidence. The corresponding IRR (IRR = 17.25; 95% CI: 8.21-41.79) and attributable fraction among the exposed (94.2%; 95% CI: 87.8-97.6%) further emphasize the substantial impact of VA type on infection risk [17]. These findings reinforce existing recommendations from the KDOQI Clinical Practice Guideline for VA to prioritize AVFs over CVCs whenever feasible [11].

The type of VA remained the strongest predictor of both BSIs and infection-related hospitalization. In the univariate Cox regression analysis, patients with a CVC had a markedly higher hazard of developing a BSI compared to those with an AVF (HR = 19.18; 95% CI: 8.72-42.23; p < 0.001) and were over four times more likely to be hospitalized due to infection (HR = 4.10; 95% CI: 2.50-6.71; p < 0.001). These results align with extensive multicenter observational studies, including the Dialysis Outcomes and Practice Patterns Study (DOPPS), which identified CVC use as the most modifiable risk factor for infection and mortality in dialysis populations.

Interestingly, neither age nor diabetes mellitus was found to be significantly linked to the risk of BSIs in this study. Although diabetes was modestly linked to a higher likelihood of infection-related hospitalization in the univariate model (HR = 1.04; 95% CI: 1.00-1.07; p = 0.041), this association did not remain significant after multivariable adjustment.

This differs from previous studies that have connected diabetes to higher infection risk in kidney failure populations [11,22]. Our results suggest that the type of VA may have a more significant impact on infection outcomes than traditional comorbidities, such as diabetes, at least in certain settings.

Despite clinical guidelines promoting AVFs as the preferred access modality [11], more than half of our patients, 208 (53.9%), relied on CVCs. This likely reflects real-world challenges such as late referral for vascular surgery, limited surgical availability, or anatomical contraindications [1]. Nevertheless, the markedly higher infection rates and associated hospitalizations among CVC users underscore the urgent need for system-level interventions to encourage early fistula creation and reduce catheter dependence.

Our study did not capture several potentially relevant covariates, including dialysis adequacy, serum albumin, and catheter dwell location or duration, which may act as unmeasured confounders. In addition, subgroup analyses, such as those stratified by diabetes or age, were limited by statistical power due to the relatively small number of infection events. These factors restrict the precision of subgroup estimates and underscore the need for cautious interpretation. Future large multicenter studies with more comprehensive covariate data will be essential to validate and extend our findings.

The strengths of this study include a well-defined cohort, a long observation period (median = 1,096 days), and comprehensive documentation of infection events and outcomes. However, several limitations must be acknowledged. The single-center design may limit generalizability, and the retrospective nature of the study introduces potential risks of incomplete or misclassified data. In addition, microbiological profiles and details of antibiotic regimens were not included in the analysis, which limits clinical interpretation.

## Conclusions

Our findings reaffirm that CVCs are a significant and modifiable risk factor for BSIs and infection-related hospitalizations in patients undergoing HD. Lower infection rates in our cohort may be associated with the use of catheter locking solutions and strict surveillance. In addition, reducing CVC use, promoting timely creation of AVFs, and enforcing standardized infection-control measures could further contribute to improved VA outcomes and patient safety.

Accurate identification of infection sources, combined with targeted prevention strategies, should be prioritized at both institutional and national levels. These findings support the implementation of multifaceted interventions, such as early AVF placement, reducing catheter dependence, and strictly adhering to evidence-based infection-prevention protocols. Such measures should be strengthened through systematic VA planning, structured patient education, and coordinated public health efforts.

Importantly, these results should be interpreted as associations consistent with the retrospective design. Larger prospective multicenter studies with standardized definitions and microbiology are required to confirm causality and provide definitive clinical guidance, particularly in healthcare systems with high CVC prevalence.
